# [Expression of Concern] Endogenous Nampt upregulation is associated with diabetic nephropathy inflammatory-fibrosis through the NF-κB p65 and Sirt1 pathway; NMN alleviates diabetic nephropathy inflammatory-fibrosis by inhibiting endogenous Nampt

**DOI:** 10.3892/etm.2026.13189

**Published:** 2026-05-20

**Authors:** Ye Chen, Yuzhen Liang, Tingting Hu, Riming Wei, Congjie Cai, Ping Wang, Lingyu Wang, Wei Qiao, Leping Feng

Exp Ther Med 14:4181–4193, 2017; DOI: 10.3892/etm.2017.5098

Following the publication of this paper, it was drawn to the Editor’s attention by a concerned reader that the Sirt1 western blotting data shown in Fig. 4A on p. 4187 were strikingly similar to the Sirtl western blotting data shown in Fig. 6A on p. 4188, even though the experimental conditions reported for these figure parts were apparently different.

The authors have been contacted by the Editorial Office to offer an explanation for the apparent re-use of the same data in this paper, and we are awaiting their response. Owing to the fact that the Editorial Office has been made aware of potential issues surrounding the scientific integrity of this paper, we are issuing an Expression of Concern to notify readers of this potential problem while the Editorial Office continues to investigate this matter further.

